# Reducing the consumption of personal protective equipment by setting up a multifunctional sampling station in the emergency department to screen for COVID-19 infection in Taiwan

**DOI:** 10.1186/s12199-020-00874-5

**Published:** 2020-07-30

**Authors:** Po-Ting Lin, Ting-Yuan Ni, Tren-Yi Chen, Chih-Pei Su, Hsiao-Fen Sun, Mu-Kuan Chen, Chu-Chung Chou, Po-Yu Wang, Yan-Ren Lin

**Affiliations:** 1 Department of Emergency and Critical Care Medicine, Changhua Christian Hospital, Changhua, Taiwan; 2 Department of Nursing, Changhua Christian Hospital, Changhua, Taiwan; 3 Department of Otorhinolaryngology, Head and Neck Surgery, Changhua Christian Hospital, Changhua, Taiwan; 4 School of Medicine, Chung Shan Medical University, Taichung, Taiwan; 5 School of Medicine, Kaohsiung Medical University, Kaohsiung, Taiwan; 6 Department of Pediatric Emergency, Changhua Christian Children’s Hospital, Changhua, Taiwan

**Keywords:** Personal protective equipment (PPE), Multifunctional sampling station, COVID-19, Emergency department

## Abstract

In Taiwan, high-risk patients have been identified and tested for preventing community spread of COVID-19. Most sample collection was performed in emergency departments (EDs). Traditional sample collection requires substantial personal protective equipment (PPE), healthcare professionals, sanitation workers, and isolation space. To solve this problem, we established a multifunctional sample collection station (MSCS) for COVID-19 testing in front of our ED. The station is composed of a thick and clear acrylic board (2 cm), which completely separates the patient and medical personnel. Three pairs of gloves (length, 45 cm) are attached and fixed on the outside wall of the MSCS. The gloves are used to conduct sampling of throat/nasal swabs, sputum, and blood from patients. The gap between the board and the building is only 0.2 cm (sealed with silicone sealant). ED personnel communicate with patients using a small two-way broadcast system. Medical waste is put in specific trashcans installed in the table outside the MSCS. With full physical protection, the personnel conducting the sampling procedure need to wear only their N95 mask and gloves. After we activated the station, our PPE, sampling time, and sanitization resources were considerably conserved during the 4-week observation period. The MSCS obviously saved time and PPE. It elevated the efficiency and capacity of the ED for handling potential community infections of COVID-19.

Dear Editor,

The outbreak of COVID-19 has devastated the community and health care facilities [1]. Nationwide extensive screening of potential COVID-19 patients might be beneficial for early identification, treatment, and the development of an isolation policy [2]. However, for some countries that are facing potential community infections and are not truly in the epidemic stage, extensive screening might rapidly consume the current personal protective equipment (PPE) and hospital capacity resources and may even result in resource insufficiency in the subsequent epidemic stage [3]. Therefore, tracing and identification of high-risk patients, especially those with positive travel, occupation, contact, and cluster (TOCC) histories, would be very useful before the epidemic stage. More importantly, rapid sample collection and examination without much PPE or hospital capacity consumption would maintain the normal functioning of emergency departments (EDs) and even the whole health care system.

In Taiwan, high-risk patients have been identified and tested (by the recommendation of the Taiwan CDC) for preventing potential COVID-19 community spread. Most of the sample collection was performed in EDs. Traditional methods for sample collection require substantial PPE, healthcare professionals, sanitation workers, and isolation space. These methods are also very time consuming, requiring wearing and removing the PPE with repeated sampling and sanitization. To solve this problem, we established a multifunctional sample collection station (MSCS) for COVID-19 testing in front of our ED. This station is composed of a thick and clear acrylic board that completely separates the patient and medical personnel. Detailed information regarding the measurements is provided in Fig. 1. Three pairs of gloves (length, 45 cm) are attached and fixed on the outside wall of the MSCS. The gloves are used to conduct sampling of throat/nasal swabs, sputum, and blood from patients. The thickness of the acrylic board on the MSCS is 2 cm. The gap between the board and building is only 0.2 cm (sealed with silicone sealant). ED personnel communicate with patients using a small two-way broadcast system (with fixed microphones and speakers on the wall). Medical waste (including alcohol-soaked cotton and tongue depressor) is placed in specific trashcans (15 cm^2^, depth 20 cm), which are installed in the table outside the MSCS. With full physical protection, the personnel who conduct the sampling procedure need to wear only their N95 mask and gloves (Fig. 2).

**Fig. 1:**
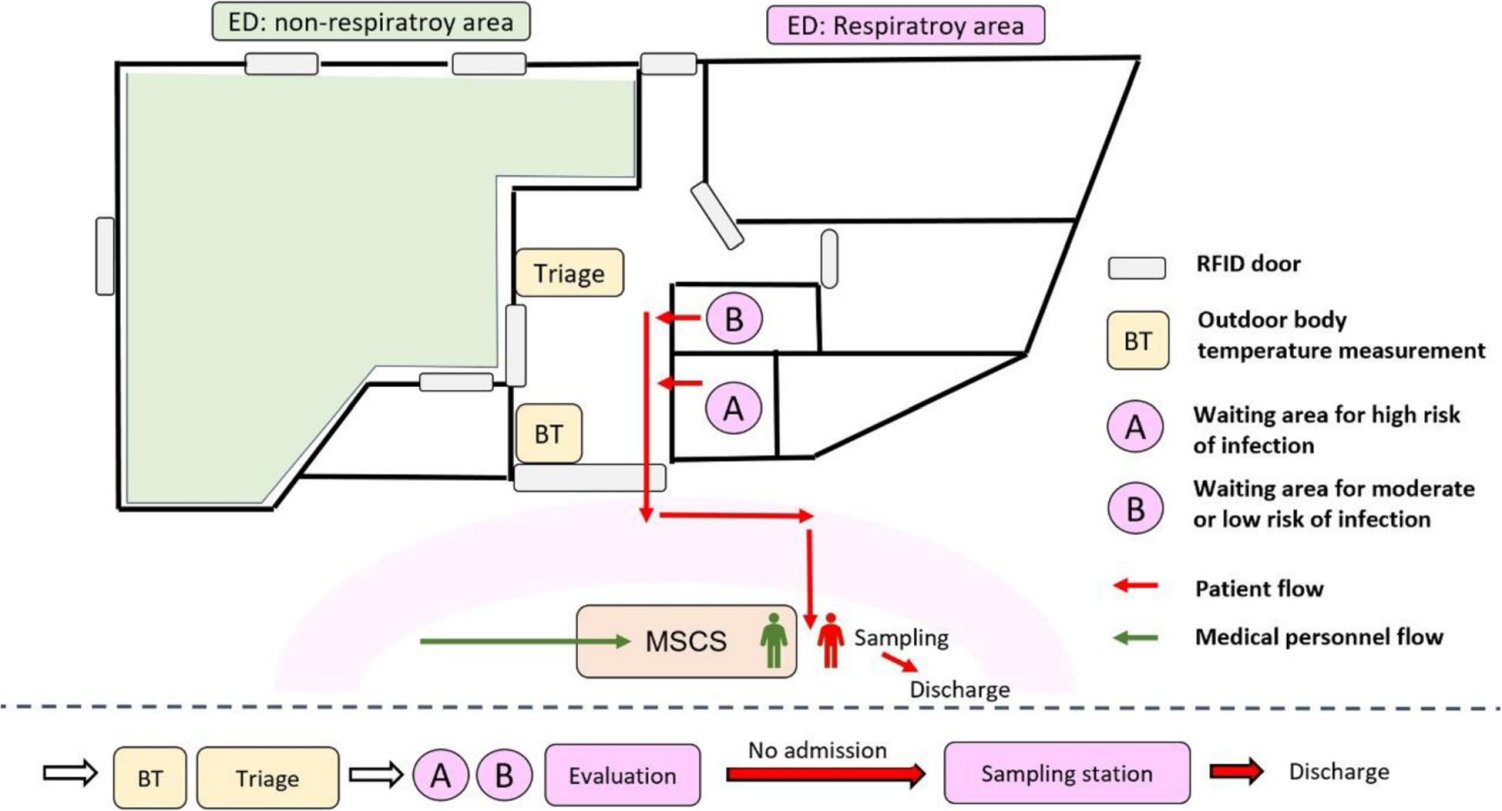
Workflow of patients and medical personnel when using a multifunctional sample collection station (MSCS). RFID, radio-frequency identification device

**Fig. 2:**
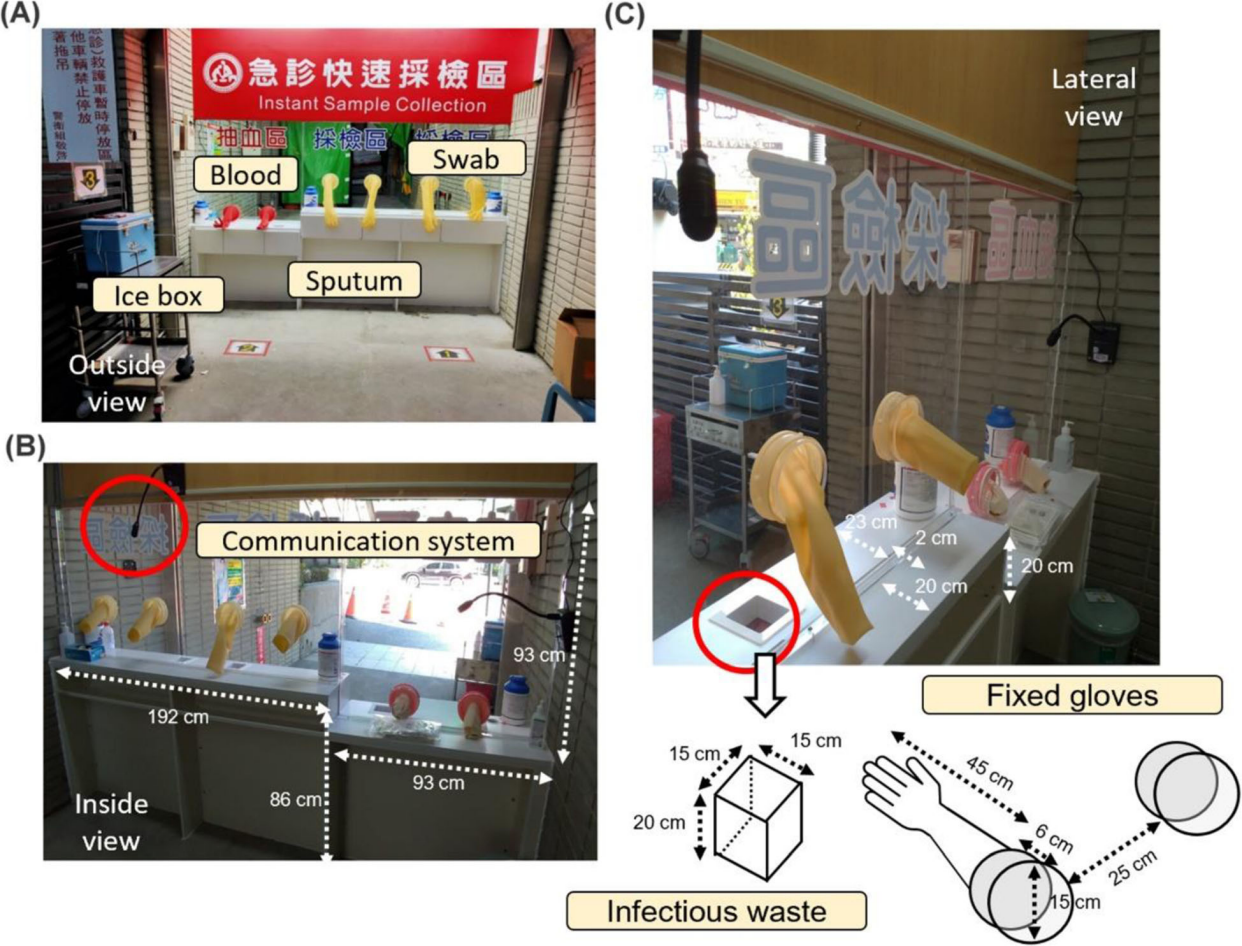
**a** Outside, **b** inside, and **c** lateral views of the multifunctional sample collection station (MSCS). Before sampling, each patient would have their personal bag (including a syringe, alcohol-soaked cotton, a swab, gloves, a tongue depressor, and a sputum collection bottle). After sampling, they put their samples in the ice box by themselves. This station serves only one patient at a time

Each ED patient rapidly received outdoor body temperature measurements and TOCC examinations conducted by machines and triage counter personnel. If they were identified as having a high/moderate risk of COVID-19 infection, they received a quick evaluation in separate risk-associated waiting areas [4]. After initial evaluation of a chest image, virus testing for COVID-19 was performed in the MSCS (including throat/nasal swab, sputum collection, blood testing for antibody). This station served only one patient at a time (reducing the risk of cross-infection). Once the patients completed the tests, they could be discharged directly from station and await their reports at home.

After we activated the station, our PPE, sampling time, and sanitization resources were considerably conserved during the 4-week observation period. A comparison between traditional sampling (in a single negative-pressure isolation room) and using the MSCS (outside of the ED) is shown in Table 1. In conclusion, the MSCS considerably saved time and PPE. It elevated the efficiency and capacity of the ED when handling potential community infections of COVID-19.

**Table 1:** Comparing the differences between traditional sampling and the multifunctional sample collection station (MSCS) during the 4-week observation period

Variables	Traditional (the first two weeks)	MSCS (the last two weeks)	Unit
The number of PPE used for sampling (median)	24	0	Per day
Time for suiting up and removal (minutes, median)	21	1	Per patient
Time for sample collection (minutes, median)	5	2	Per patient
Time for sanitization (minutes, median)	35	10	Per patient

## Data Availability

Not applicable.

## References

[1] Mareiniss DP. The impending storm: COVID-19, pandemics and our overwhelmed emergency departments. Am J Emerg Med. 2020 Mar 23. pii: S0735-6757(20)30175-3. doi: 10.1016/j.ajem.2020.03.033. 10.1016/j.ajem.2020.03.033 PMC7102611 32253132

[2] World Health Organization. Home care for patients with suspected novel coronavirus (nCoV) infection presenting with mild symptoms and management of contacts. WHO reference number: WHO/nCov/IPC/HomeCare/2020.3. Published February 4, 2020. Accessed February 24, 2020.

[3] Hick JL, Biddinger PD. Novel coronavirus and old lessons — preparing the health system for the pandemic. N Engl J Med. 2020 Mar 25. 10.1056/NEJMp2005118. 10.1056/NEJMp2005118 32212515

[4] Chen TY, Lai HW, Hou IL, Lin CH, Chen MK, Chou CC, Lin YR (2020). Buffer areas in emergency department to handle potential COVID-19 community infection in Taiwan. Travel Med Infect Dis..

